# Isolation and identification of dark septate endophytes from sorghum roots and the effects of *Alternaria destruens* HN16G1 on drought and low-nutrient tolerance in sorghum seedlings

**DOI:** 10.3389/fmicb.2026.1829731

**Published:** 2026-05-22

**Authors:** Yali Zhang, Zhenfeng Gao, Huilin Long, Rong Wang, Wenbin Bai

**Affiliations:** 1 College of Resources and Environment, Shanxi Agricultural University, Taigu, China; 2 Soil Health Laboratory in Shanxi Province, Shanxi Agricultural University, Taiyuan, China; 3 College of Food Science and Engineering, Shanxi Agricultural University, Taiyuan, China; 4 Sorghum Research Institute, Shanxi Agricultural University, Jinzhong, China

**Keywords:** dark septate endophytes, drought tolerance, growth-promoting traits, salt tolerance, sorghum

## Abstract

**Introduction:**

Dark septate endophytes (DSE) are root-associated fungi that can enhance host stress tolerance, yet their diversity and function in sorghum remain unclear. Here, we investigated the culturable DSE community across 29 sorghum varieties and evaluated the symbiotic potential of key isolates.

**Methods:**

Culturable DSE were isolated from sorghum rhizosphere soil and roots via plate isolation, microscopy, and colonization assessment to screen efficient colonizers. Pot experiments then analyzed symbiotic compatibility with major Chinese cultivars to identify strains with high colonization efficiency. Finally, the selected strain was evaluated for salt tolerance and its effects on drought resistance, growth promotion, and low-fertility tolerance in seedlings.

**Results and discussion:**

A total of 94 DSE isolates were obtained, accounting for 30.72% of the total fungal isolates, and were classified into 30 morphotypes. The genus *Alternaria* emerged as the dominant group, comprising 46.67% of the isolates. Among them, strain HN16G1, isolated from Hongnuo 16 and identified as *Alternaria destruens* via internal transcribed spacer sequencing, demonstrated superior root colonization capability. Strain HN16G1 established stable symbiosis with all 10 commercial cultivars tested, maintaining colonization rates and intensities above 50.0 and 16.0%, respectively. Physiologically, HN16G1 exhibited moderate halotolerance, with low NaCl concentrations (0.1–0.2 mol/L) promoting mycelial growth, whereas high salt stress (0.6 mol/L) induced adaptive responses including enhanced sporulation and reduced hyphal septal distance. The strain also displayed substantial indole-3-acetic acid production, reaching 181.56 μg/mL. Inoculation with HN16G1 (5.0 × 10^5^ CFU/mL) significantly improved seedling emergence and early vegetative growth in sorghum under combined drought stress (40% field capacity) and low-nutrient conditions. Notably, varieties “Hongnuo 16” and “Jinnuo 3” exhibited the most pronounced responses, with growth parameters approaching optimal levels. Colonization parameters (rate and intensity) correlated positively with plant height, root length, and biomass. Overall, these findings highlight the rich DSE diversity in sorghum roots and identify *A. destruens* HN16G1 as a broad-compatibility, multifunctional endophyte with promising potential for the development of microbial inoculants to enhance stress tolerance in sorghum production systems.

## 1 Introduction

Sorghum is the fifth largest cereal crop in the world after wheat, rice, maize, and barley (Khaskheli et al., 2025; Zahid et al., 2025). As an important food, forage, and energy crop, it exhibits resistance to multiple abiotic stresses, including drought, salt-alkali, poor soil, and waterlogging (Mukherjee et al., 2024; Katamreddy et al., 2025; Khaskheli et al., 2025). Although sorghum is a potential “star” crop for marginal farmland and medium- to low-quality farmland in China, drought, soil salinization, decline in soil fertility, and continuous cropping obstacles remain the core constraints limiting high and stable yields in the main sorghum-producing regions of China (Gao F. C. et al., 2022; Yan et al., 2023). To enhance the stress resistance of sorghum, overcome continuous cropping challenges, and improve soil fertility, in recent years, stress-resistant cultivar screening (drought-tolerant and salt-alkali-tolerant varieties) (Goche et al., 2020; Ren et al., 2022; Yahaya et al., 2023; Qiao et al., 2025), soil improvement (microbial community optimization) (Wu et al., 2019; Xue et al., 2024), crop rotation design (Shanker et al., 2023; Li G. et al., 2024), fertilizer technology (Zhou et al., 2024; Wang R. et al., 2025), and rhizosphere autotoxic substances (Tibugari et al., 2020; Tibugari and Chiduza, 2024) have been extensively explored. Stress-resistant and growth-promoting microorganisms have also been proven to be effective in promoting crop stress resistance and high yield in wheat (Elhaissoufi et al., 2024; Liu et al., 2024; Malik et al., 2024; Ye et al., 2025), maize (Gong et al., 2023; Fan et al., 2024; Wang Y. et al., 2024), tomato (Zhu et al., 2018; Karačić et al., 2024; Wei et al., 2024), potato (Das and and Kayang, 2010; Shi et al., 2024; Liu et al., 2025), buckwheat (Gao Y. et al., 2022; Zhong et al., 2025), and millet (Swamy, 2023; Umashankar et al., 2023). Moreover, the effects of applying plant growth-promoting rhizobacteria (PGPR), arbuscular mycorrhizal fungi (AMF), and dark septate endophytes (DSE) in production are widely recognized (Malicka et al., 2022; Hnini et al., 2024; Huertas et al., 2024; Jian et al., 2024; Mariani et al., 2024; Sun et al., 2024). However, current exploration and utilization of stress-resistant and growth-promoting microbial resources in sorghum have predominantly focused on PGPR and AMF, with functionalities, such as salt-alkali tolerance, heavy metal resistance, and phosphate-solubilizing capacities (El-Meihy et al., 2019; Chandra et al., 2022; Abbasi et al., 2024; Aliyat et al., 2024; Chen et al., 2024; Rajabi Dehnavi et al., 2024), remaining underdeveloped. Applied studies on stress-resistant and growth-promoting microorganisms, especially the types of DSE that coexist with sorghum and their application effects in sorghum cultivation, remain unclear compared with that in crops such as wheat, corn, and tomato.

Dark septate endophytes (DSE)—a category of stress-tolerant microorganisms—are endophytic fungi that primarily colonize intracellular spaces and intercellular regions within the root tissues of host plants. They are characterized by dark septate hyphae and microsclerotia as their main colonization structures (Hidayat, 2019; Santos et al., 2021; Mariani et al., 2024). DSE not only enhance host plant growth by promoting nutrient absorption and utilization but also colonize harsh environments, such as drought, salinization, and heavy metal-contaminated soils, thereby improving the stress resistance of host plants and demonstrating significant ecological functions (Akhtar et al., 2022; Malicka et al., 2022; Netherway et al., 2024). DSE have been reported as critical microbial resources, besides mycorrhizal fungi, capable of effectively enhancing crop disease tolerance and nutrient absorption efficiency (Vergara et al., 2017; Surono and Narisawa, 2018; Harsonowati et al., 2020; Xu et al., 2020). Consequently, these endophytes have acquired considerable research attention, with a focus on enhancing crop stress resistance. The beneficial effects of DSE application have been confirmed in many crops; for example, the DSE strain *Alternaria alstroemeriae* could enhance drought tolerance of *Isatis indigotica* and improve its epigoitrin content (Li et al., 2023), *Falciphora oryzae* conferred improved Cd tolerance to rice via *SNARE Syntaxin 1*-regulated chlamydospore formation and vacuole enlargement (Su et al., 2021), *Alternaria* sp. 17,463 could enhance soil structure and root development in alfalfa (Bi et al., 2025b), *Exophiala pisciphila* GM25 isolated from maize could enhance drought tolerance of sorghum seeding (Zhang et al., 2017), *Cladophialophora chaetospira* SK51 could enhance *Fusarium* wilt resistance of strawberry (Harsonowati et al., 2020), and *Alternaria chlamydosporigena* improved salt tolerance of *Artemisia ordosica* (Hou et al., 2021). In addition, interaction of DSE with AMF, impact of ecological environment on their distribution, biological effects of their metabolites, resistance to chemical pesticides, and mechanisms through which they enhance crop stress resistance have been studied in different crop (Scervino et al., 2009; Spagnoletti and Chiocchio, 2020; Wu et al., 2021; Wang et al., 2022; Chen et al., 2023; Wang et al., 2023; Wang Z. et al., 2024; Wang Z. et al., 2025). However the survival status of DSE in the sorghum rhizosphere and whether they perform specific functions in sorghum remains unclear.

In this study, we aimed to characterize the distribution differences of DSE across different sorghum cultivars, to identify dominant strains with high colonization efficiency, and to evaluate the biological characteristics of the selected highly efficient colonizing strain.

## 2 Methods

### 2.1 Isolation and microscopic identification of dark septate fungi from different sorghum varieties

#### 2.1.1 Sample collection

The rhizosphere soil and root tissue samples of different sorghum varieties from different ecological regions were collected through the methods described by Peng et al. (2021) and Potshangbam et al. (2017), respectively. The rhizosphere soil and root tissue samples of the sorghum varieties “Han qing 3,” “Hong nuo 8,” “Hong nuo 11,” “Hong nuo 13,” “Hong nuo 16,” “Hong nuo18,” “Hong nuo 19,” “Hong nuo 25,” “Hui za 23,” “Jin nuohong 10,” and “Sui za 9” were collected from the sorghum plant base in Xiu wen Village, Jinzhong City, Shanxi Province (112° 43′5.117″E, 37° 36′37.796″N). The rhizosphere soil and root tissue samples of the sorghum varieties “Jin za 22,” “Jin za 2001,” “Jin liang 3,” “Jin liang 208,” “Jin nuoliang 9,” “Jin zao 5577,” “Liao za 36,” “Jin za 51,” “Jin Bainuo 1,” and “Jin za 28” were collected from the sorghum plant base in Xi shuangshan Village, He shengbao Township, Shan yin County, Shuo zhou City, Shanxi Province (112° 56′18.143″E, 39° 35′12.185″N). The rhizosphere soil and root tissue samples of the sorghum varieties “Jin nuo 3,” “Liao nuo 11,” “Jin za 5564,” and “Jin za 21–1” were collected from the sorghum plant base in Duanliu Village, Duanliu Township, Qinxian County, Changzhi City, Shanxi Province (112° 43′18.851″E, 36° 42′47.880″N). The rhizosphere soil and root tissue samples of the sorghum varieties “Jin nuoliang 10,” “Jin za 99,” “Liao za 52,” and “Liao nuo 10” were collected from the sorghum plant base in Nantao Village, Lucheng District, Changzhi City, Shanxi Province (113° 22′57.562″E, 36° 18′11.578″N).

#### 2.1.2 Isolation and microscopic identification of dark septate fungi

The potato dextrose agar (PDA) medium (20% potato, 2% glucose, and 0.012% streptomycin) was used for isolation and purification of fungi. The dark septate fungi present in different rhizosphere soil samples were isolated through method B described previously (Kawaguchi et al., 2012), with slight modifications. For each variety, 4 g of rhizosphere soil was placed in a 250 mL Erlenmeyer flask containing 100 mL of sterile water and shaken at 180 r/min and 25 °C for 30 min to prepare a soil suspension. The suspension was serially diluted to 10^−3^, 10^−4^, 10^−5^, 10^−6^, and 10^−7^. From each dilution, 100 μL was spread onto PDA plates (90 mm in diameter) supplemented with 100 μg/mL streptomycin sulfate. The plates were incubated inverted at 26 °C for 7 days. After colonies with distinct morphological characteristics appeared on the plates, mycelia from colony margins showing differing in color, morphology, and growth rate were picked using an inoculation needle and transferred to fresh PDA plates for purification. Subculturing was repeated until pure cultures with uniform morphology were obtained. Isolation was performed in triplicate for each sample. Pure cultures with obvious differences in colony color, morphology, and growth rate were selected and stored at 4 °C for subsequent use.

The dark septate fungi from different root tissue samples were isolated via methods described by Potshangbam et al. (2017), with slight modifications. Five roots were randomly selected from each variety. From each root, 2–3 cm segments were collected from the upper, middle, and lower regions and cut into 0.5 cm pieces. After washing with sterile water, the segments were surface-sterilized with 1.5% NaClO for 1 min and 75% ethanol for 1 min under aseptic conditions, followed by three rinses with sterile water. The samples were then blotted dry and placed onto PDA plates supplemented with 100 μg/mL streptomycin sulfate (5 pieces per plate). Plates were incubated at 26 °C in the dark for 7 days. Once mycelia emerged from the tissue segments, hyphae from colonies differing in color and morphology were picked and transferred to fresh PDA plates for purification until pure cultures were obtained. Three replicates were performed for each sample. Pure cultures with distinct differences in color, morphology, and growth rate were stored at 4 °C.

The putative dark septate fungi were preliminarily identified by microscopic observation based on mycelial pigmentation and septation, and subsequently stored at 4 °C for future analysis.

### 2.2 Screening of DSE

The DSE were selected from among the dark septate fungi, isolated as described in section 2.1, using a colonization test in sorghum.

#### 2.2.1 Preparation of fungal suspension

All dark septate fungi were inoculated in 1,000 mL Erlenmeyer flasks containing 800 mL of potato dextrose broth (PDB) by transferring a 5-mm diameter fungal plug punched out from PDA plates after being activated by culturing for 5 days. All dark septate fungi were then cultured for 7 days at 28 °C with shaking at 170 rpm. The spore suspension was collected by filtering the mycelia through four layers of sterile gauze to remove mycelial fragments, and the spore concentration was adjusted to 5.0 × 10^5^ colony forming units (CFU)/mL with sterile distilled water.

#### 2.2.2 Determination of the colonization rate

Sorghum varieties were selected based on the sources of DSE isolation. The seeds were surface-sterilized by soaking in 1.5% sodium hypochlorite solution for 10 min, followed by three consecutive rinses with sterile distilled water. After sterilization, 50 plump and uniform seeds were selected and transferred into sterile Petri dishes containing 10 mL of fungal suspension. Sterile water was used as the non-inoculated control (CK). All treatments were performed in triplicates.

The colonization structures of DSE were stained following a previously described method (Liu et al., 2018), withslight modifications. On the 5th day of co-culture, approximately 1.5 g of fresh sorghum roots were randomly collected, washed three times with sterile water, cut into 1 cm segments, and placed into embedding cassettes. The samples were treated with 10% KOH at 90 °C for 60 min, rinsed three times with sterile water, then soaked in 2% HCl at room temperature for 8 min. After rinsing again, the samples were stained with 0.05% trypan blue at 90 °C for 30 min. Following three additional rinses, samples were destained in a lactic acid:glycerol:water (1:1:1) at room temperature for 2 days. After destaining, 30 root segments were randomly selected, mounted (10 per slide), and examined under a microscope. Colonization rate and colonization intensity of DSE in sorghum roots were calculated using the following Equations 1 and 2 (Biermann and Linderman, 1981). All treatments were performed in triplicates.

$$\text{Colonization rate}\%=\frac{\text{Number of infected root segments}}{30}\times 100\%$$ (1)

$$\text{Colonization intensity}\%=\frac{\sum_{1-30}\frac{\text{Colonized length}\;\mathrm{per}\;\text{root segment}}{\text{Total length}\;\mathrm{per}\;\text{root segment}}}{\text{Total number of colonized root segments}}\times 100\%$$ (2)

#### 2.2.3 Analysis of differential colonization of DSE across sorghum cultivars

To screen for DSE capable of efficient colonization across diverse sorghum varieties, 10 commercially important cultivars (“Jinza 22,” “Hongnuo 16,” “Jinnuo 3,” “Jinza 2001,” “Jinbainuo 1,” “Jiza 163,” “Jinza 18,” “Jinzao 5577,” “Liaoza 19,” and “Yinuohong 9”) were selected to assess DSE colonization rates. The methodology for assessing DSE colonization rates is detailed in section 2.2. All treatments were conducted in triplicates.

#### 2.2.4 Identification of the internal transcribed spacer sequence of the DSE HN16G1

The genomic DNA of DSE HN16G1 was extracted following the method established by Osmundson et al. (2013). PCR amplification was performed using the ITS1 (5′-TCCGTAGGTGAACCTGCGG-3′) and ITS4 (5′-TCCTCCGCTTATTGATATGC-3′) primers (Bokulich and Mills, 2013). The reaction mixture (25 μL) contained 12.5 μL Premix Taq™ (TaKaRa Taq™ Version 2.0), 0.5 μL each of primers ITS1 and ITS4, 0.5 μL genomic DNA, and 11 μL ddH_2_O. The thermocycling parameters were as follows: initial denaturation at 94 °C for 5 min, 30 cycles of denaturation at 94 °C for 45 s, annealing at 55 °C for 40 s, and extension at 72 °C for 30 s, followed by final extension at 72 °C for 10 min. PCR products that passed quality control via electrophoresis were sent to Beijing Liuhe BGI for sequencing.

The internal transcribed spacer (ITS) sequence of DSE HN16G1 was first aligned with those of type strains using the online BLAST tool available at NCBI website. Subsequently, ITS gene sequences from closely related type strains were selected, and manually trimmed to remove ambiguous ends, and aligned using Clustal W in MEGA 5.0. A neighbor-joining (NJ) phylogenetic tree was constructed using the MEGA 5.0 software to determine the taxonomic relationship among the strains.

### 2.3 Analysis of the biological functions of the DSE HN16G1

#### 2.3.1 Analysis of salt tolerance characteristics of HN16G1

To simulate saline–alkali stress, different concentrations of salts were added to PDA medium. Five salt concentration gradients (0, 0.1, 0.2, 0.4, and 0.6 mol/L) of HN16G1 were established (Gonzalez Mateu et al., 2020). The experiment and each treatment were replicated thrice.

Under sterile conditions in a clean bench, mycelial plugs (diameter, 8 mm) were obtained from HN16G1 colonies that had been activated on PDA plates for 8 days. These plugs were then inoculated onto PDA plates with different saline-alkali concentrations. The cultures were maintained at 26 °C for 8 days, with colony diameters measured every 2 days using a ruler. The saline–alkali tolerance characteristics of HN16G1 were evaluated based on growth rate and final colony diameter after 8 days of cultivation.

#### 2.3.2 Quantitative assay of IAA secretion capacity of HN16G1

IAA Secretion Assay: A mycelial plug (diameter, 8 mm) of HN16G1 was inoculated into a 300-mL Erlenmeyer flask containing 150 mL PDB fermentation medium supplemented with 0.30 g/L L-tryptophan. The culture was incubated at 26 °C with shaking at 180 rpm for 7 days. After incubation, 1 mL of the fermentation broth was centrifuged at 12,000 rpm at 25 °C for 10 min. Subsequently, 500 μL of the supernatant was transferred to a clean centrifuge tube, mixed with 1,000 μL Salkowski reagent (prepared by mixing 10 mL of 0.5 mol/L FeCl_3_ solution and 500 mL of 35% perchloric acid, and stored in the dark), and allowed to react in the dark for 30 min through the method described by Guardado-Fierros et al. (2024). A color change to red indicated IAA production. The absorbance at 530 nm was measured using a spectrophotometer, and the IAA concentration was calculated based on a standard curve. The Blank control contained 500 μL sterile water and an equal volume of the Salkowski reagent, whereas the Positive control contained 500 μL IAA standard solution (50 μg/mL) and an equal volume of the Salkowski reagent.

IAA Standard Curve: IAA standard solutions were prepared at 0.5, 1.0, 5.0, 10.0, 15.0, and 20.0 mg/L. After color development with the Salkowski reagent, absorbance was measured at 530 nm (zeroed with distilled water + reagent). The standard curve was generated by plotting absorbance against concentration, yielding the linear regression equation: y = 0.0021x + 0.0054* (*R*^2^ = 0.9998).

#### 2.3.3 Effect of HN16G1 on drought tolerance in sorghum seedlings

Pot experiment design (Zhang et al., 2017; Carlson et al., 2020; Barresi et al., 2022; da Silva Santos et al., 2023): Five main sorghum varieties (“Jin za 22,” “Hong nuo 16,” “Jin nuo 3,” “Jin za 2001,” and “Jin bainuo 1”) in Shanxi production were selected as test materials to determine the effects of the HN16G1 on drought tolerance in sorghum seedlings via root inoculation in pot experiments. Pots (height: 13.5 cm; outer diameter: 18.0 cm; inner diameter: 15.5 cm; bottom diameter: 10.5 cm) were filled with 3.0 kg of sterile soil (sieved through a 2-mm mesh and sterilized at 121 °C for 30 min), mixed with basal fertilizer (0.15 g/kg KH_2_PO_4_ and 0.15 g/kg CO(NH_2_)_2_). Twelve seeds of each sorghum variety, disinfected with 1.5% sodium hypochlorite for 10 min and rinsed three times with sterile water, were sown in each pot. At 0, 2, and 4 days after sowing, 200 mL of HN16G1 spore suspension (5.0 × 10^5^ CFU/mL) was applied to the roots. Soil moisture was maintained at 40% field capacity using a weighing method. Control groups included: CK1 (treated with an equal volume of sterile water, 40% field capacity) and CK2 (70% field capacity). The experiment and each treatment were replicated three times.

Pot Management and Indicator Measurements: Seedling emergence rates were recorded 10 days after sowing. At the three-leaf stage, seedlings were thinned to six plants per pot. After 30 days, the aboveground (plant height, stem diameter, fresh weight, dry weight) and belowground (root length, fresh root weight, and dry root weight) parameters were measured. The colonization rate of HN16G1 in each treatment was also determined following the method described in Section 2.2.2. Correlation analysis was performed using the Metware Metabolism Cloud Platform^1^ to evaluate the relationships between the colonization rate and intensity of strain HN16G1 and sorghum growth phenotypes, based on pot experiment data from different varieties and treatments.

#### 2.3.4 Effect of the HN16G1 on low-fertility tolerance in sorghum seedlings

The pot experiment design was based on the protocol described in section 2.3.3, with modifications to the basal fertilizer application (Abbaszadeh-Dahaji et al., 2020; Sahib et al., 2020; Li et al., 2021): KH_2_PO_4_ (0.094 g/kg) and CO(NH_2_)_2_ (0.064 g/kg) were added to the soil. Pot management, indicator measurements, and the methods for determining colonization rate, colonization intensity, and correlation analysis were consistent with those described in Section 2.3.3.

### 2.4 Statistical analysis

The data are expressed as means of values from independent experiments ± standard errors (SE). One-way analysis of variance (ANOVA) was conducted using SPSS 17.0, with statistical significance set at *p* < 0.05.

## 3 Results

### 3.1 Differences in the distribution of DSE among different sorghum varieties

**Figure 1:**
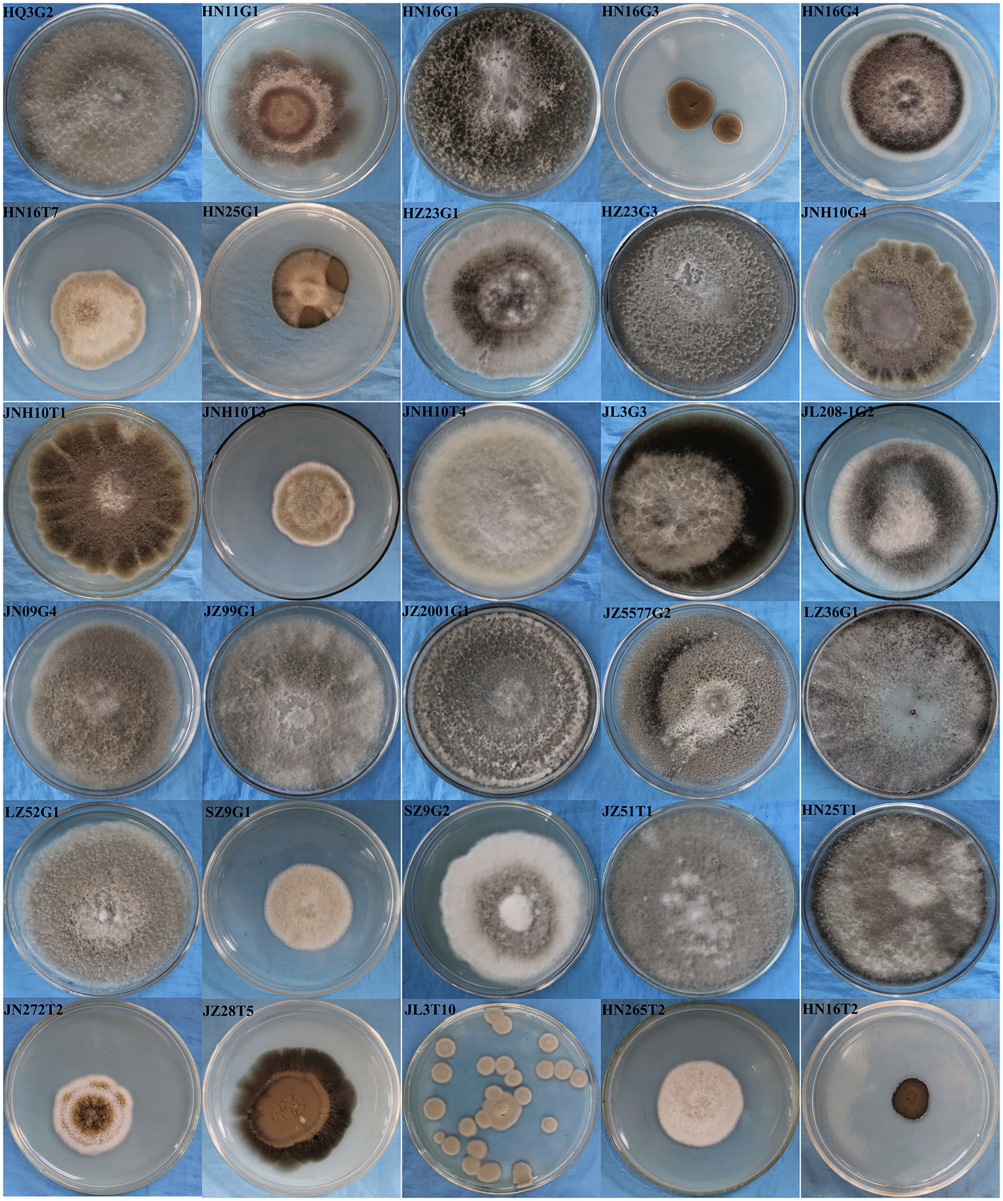
Colony morphologies of 30 dark septate fungi cultured at 26 °C for 7 days on potato dextrose agar plates.

**Figure 2:**
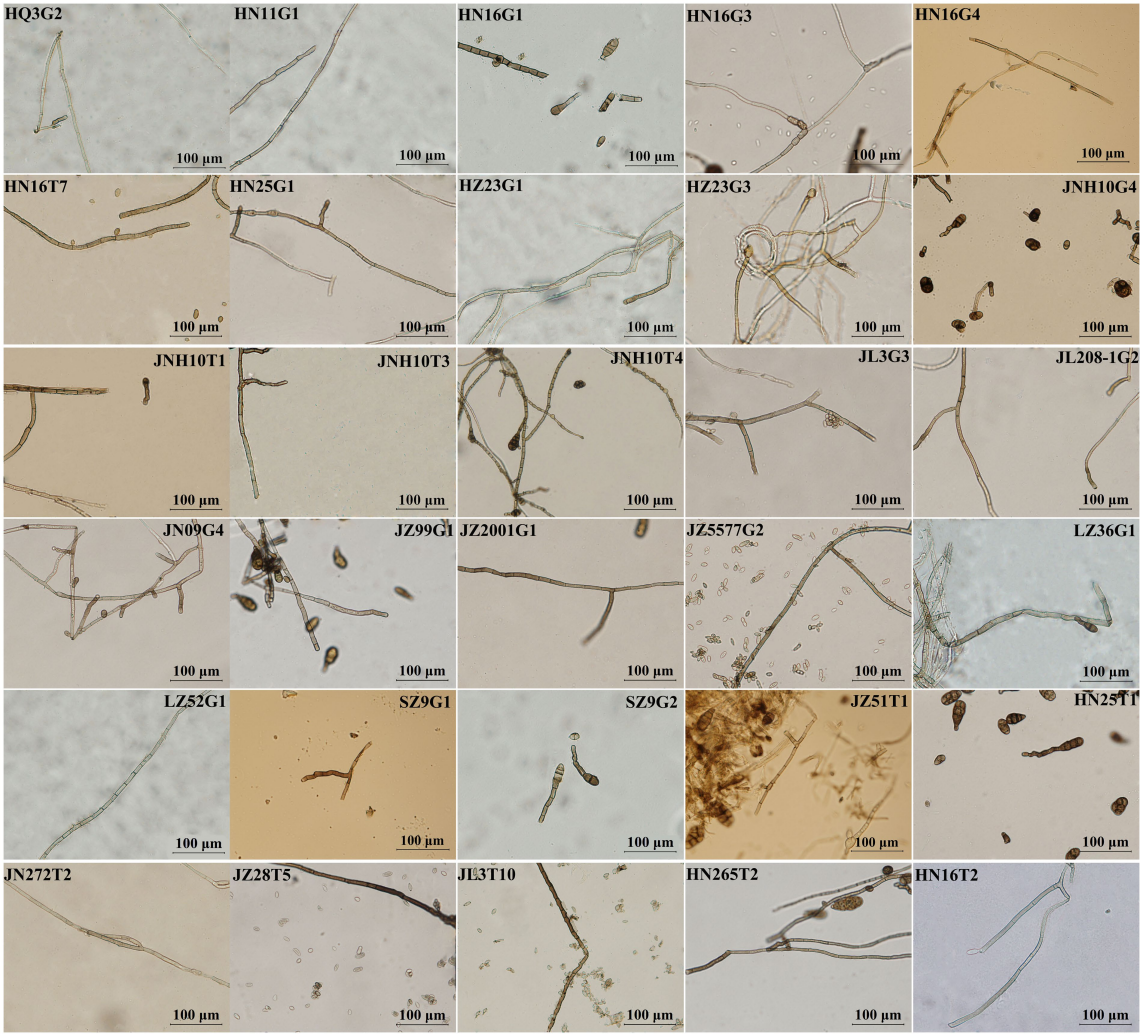
Hyphal morphologies of 30 dark septate fungi.

A total of 199 and 107 fungal strains was isolated and purified from rhizosphere soil and root tissues of 29 different sorghum varieties, respectively. Among the 306 fungal isolates, microscopic observation identified 94 as DSE, constituting 30.72% of the total isolated fungi. These endophytes were distributed across 24 distinct sorghum varieties and exhibited 30 different colony morphotypes on PDA plates (Supplementary Table S1; Figures 1, 2).

Among the 29 different sorghum varieties, the highest number of DSE (18 strains) was found in Hongnuo 16, followed by Jinza 2001 (8 strains) (Supplementary Table S1). The distribution of the 30 distinct morphotypes of dark septate fungi also varied across sorghum varieties. Among them, the colony morphology of HN16G1 was isolated from eight different sorghum varieties, and that of JNH10T1 was isolated from six varieties. The colony morphologies of HN16G4, HN16T7, JNH10G4, and JL3G3 were isolated from five different varieties each (Supplementary Table S2). Comparison of colony and hyphal morphologies among the 30 dark septate fungal isolates (Figure 1) revealed that HQ3G2, HN16G1, HN16G4, HZ23G1, HZ23G3, JNH10G4, JNH10T1, JL3G3, JL208G2, JN09G4, JZ99G1, JZ2001G1, JZ5577G2, and HN25T1 exhibited similarities to the reported colony characteristics of fungi belonging to the genus *Alternaria*, accounting for 46.67% of all isolates. This indicates that dark septate fungi of the genus *Alternaria* had a relatively high isolation frequency across different sorghum varieties and may represent the dominant group of DSE in the rhizosphere micro-ecological environment of sorghum.

**Figure 3:**
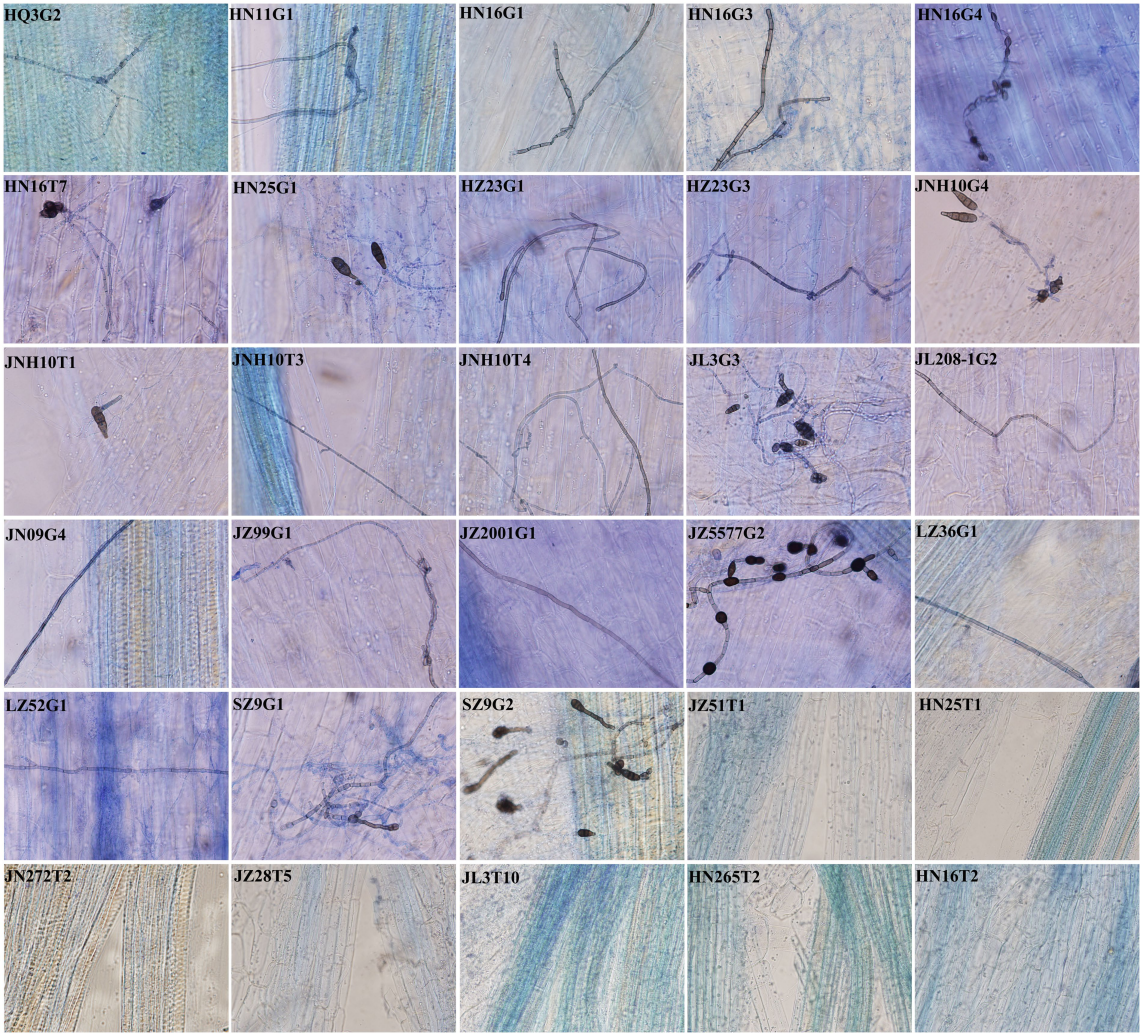
Colonization status of 30 dark septate fungi on isolated sorghum varieties.

**Figure 4:**
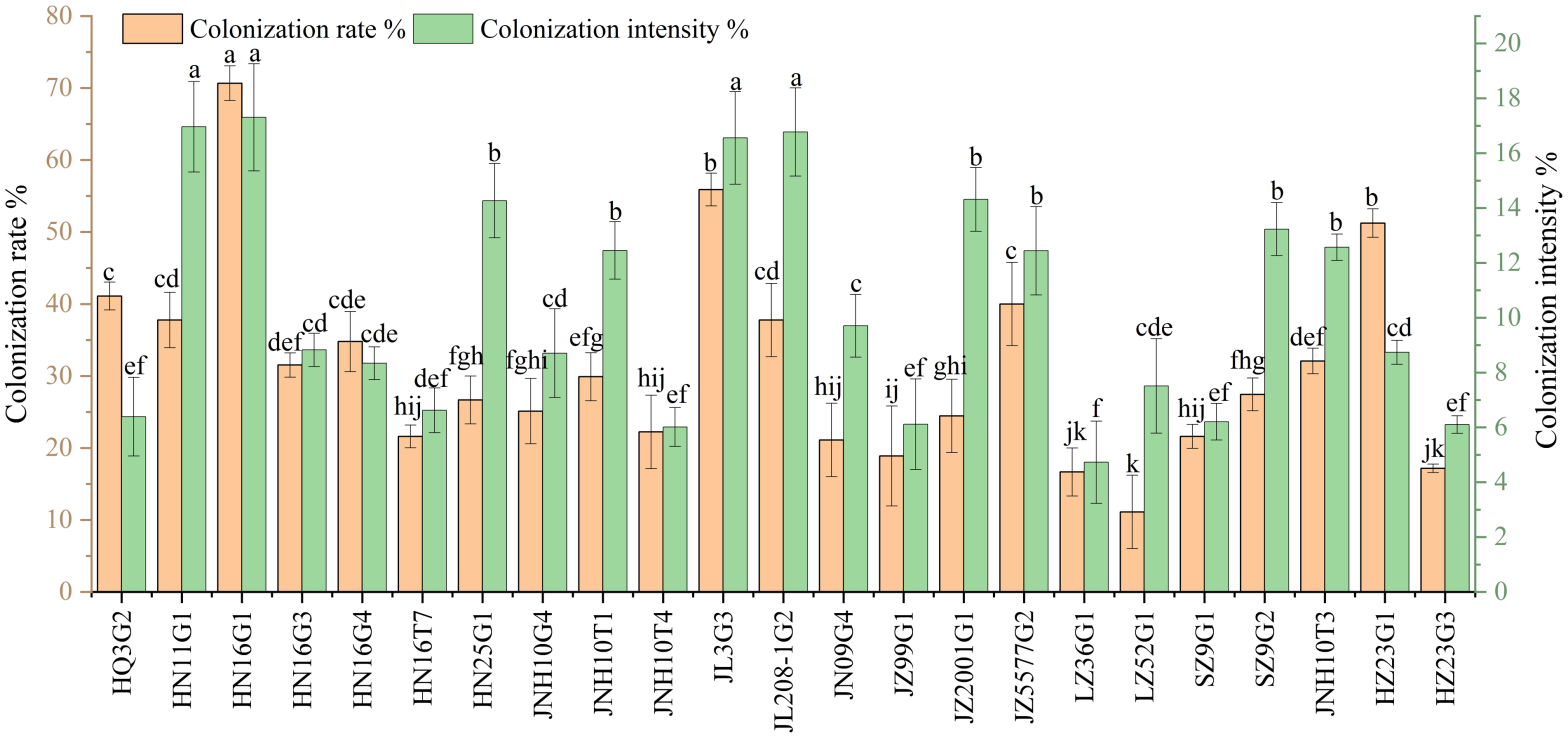
Differences in the rate and intensity of colonization of various dark septate endophytes (DSE).

Assay for colonization of sorghum root tissues by 30 morphologically distinct dark septate fungi revealed that among the 30 fungal strains, 23 were capable of infecting sorghum root tissues and were identified as DSE (Figure 3). Among them, HN16G1, JL3G3, and HZ23G1 exhibited significantly higher colonization rates, reaching 70.66, 55.89, and 51.23%, respectively, in their respective host plants, compared with other DSE. HN16G1, HN11G1, JL208-1G2, and JL3G3 showed colonization intensity values of 17.31, 16.96, 16.77, and 16.56%, respectively (Figures 3, 4), which were significantly higher than those of other strains. Based on colonization rate and colonization intensity, HN16G1 demonstrated markedly superior colonization efficacy in sorghum roots compared to the other strains tested.

### 3.2 Differences in the colonization characteristics of DSE among different sorghum cultivars

**Figure 5:**
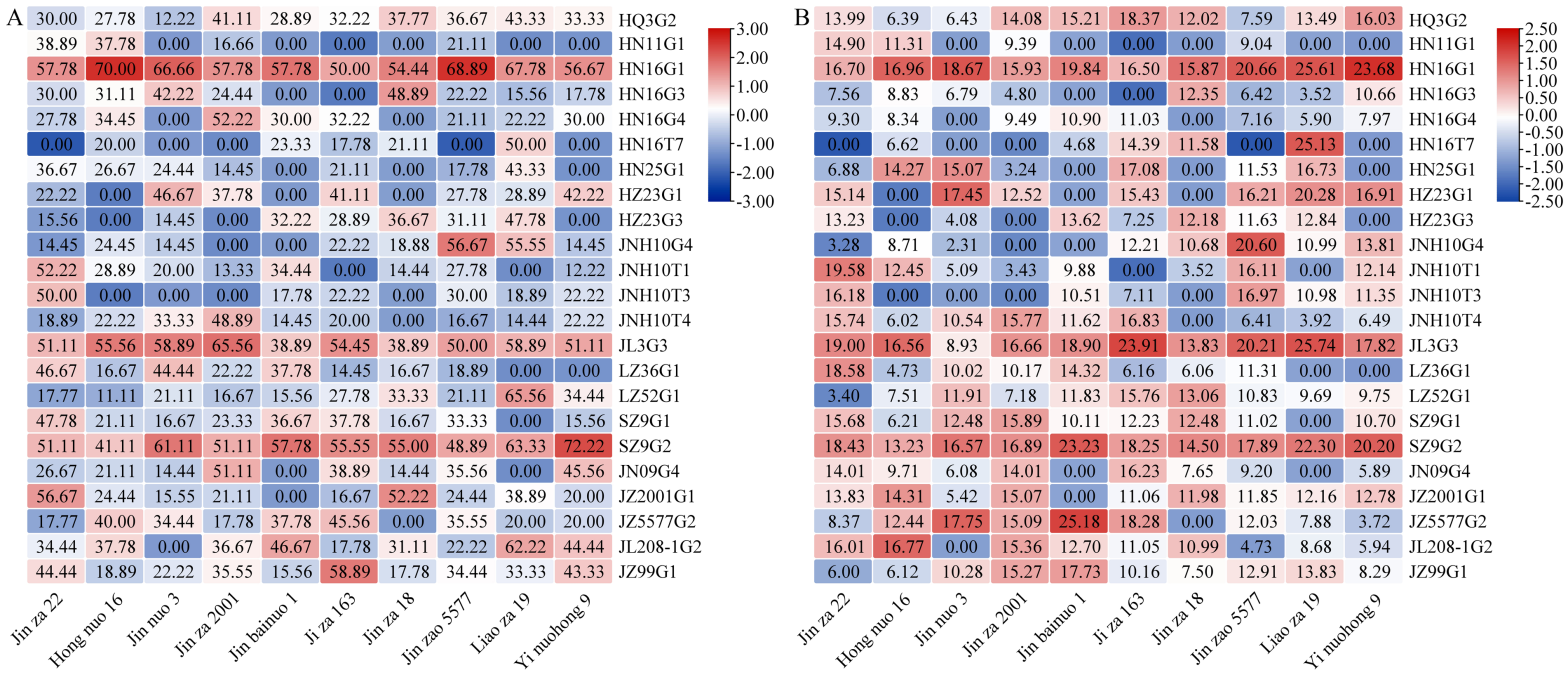
Differences in the rate **(A)** and intensity **(B)** of colonization of 23 dark septate endophytes across different sorghum varieties. Data in the figure represent means of values from three independent experiments.

To clarify the symbiotic compatibility between 23 strains of DSE and different sorghum cultivars, 10 widely cultivated varieties were selected for assessing the colonization rate. “Jin za 22,” “Hong nuo 16,” “Jin nuo 3,” “Jin za 2001,” “Jin bainuo 1,” “Ji za 163,” “Jin za 18,” “Jin zao 5577,” “Liao za 19,” and “Yi nuohong 9” could be colonized by 22, 20, 18, 16, 16, 20, 16, 22, 18, and 18 DSE strains, respectively. Among them, HQ3G2, HN16G1, JL3G3, LZ52G1, SZ9G2, and JZ99G1 were capable of colonizing all 10 tested sorghum cultivars. In contrast, HN11G1 exhibited poor symbiotic compatibility with most cultivars, colonizing only “Jin za 22,” “Hong nuo 16,” and “Jin zao 5577” (Supplementary Table S3; Figure 5).

Among the 23 DSE strains, HN16G1 achieved colonization rates and colonization intensity values above 50.0 and 16.0%, respectively, across all 10 sorghum varieties (Figure 5; Supplementary Figure S1). While strain SZ9G2 showed a notably higher rate of colonization on “Yi nuohong 9” (72.23%), compared with HN16G1, its rates of colonization on “Hong nuo 16” and “Jin zao 5577” were below 50%, with colonization intensity also lower than that of HN16G1. For JL3G3, colonization rates were below 50.0% only on “Jin bainuo 1” and “Jin za 18,” and the colonization intensity was below 16.0% only on “Jin nuo 3” and “Jin za 18”; on all other cultivars, the two parameters exceeded 50.0 and 16.0%, respectively. These findings indicate that the DSE strain HN16G1 exhibits strong symbiotic compatibility with the sorghum cultivars tested in this study.

### 3.3 ITS sequence identification of HN16G1

**Figure 6:**
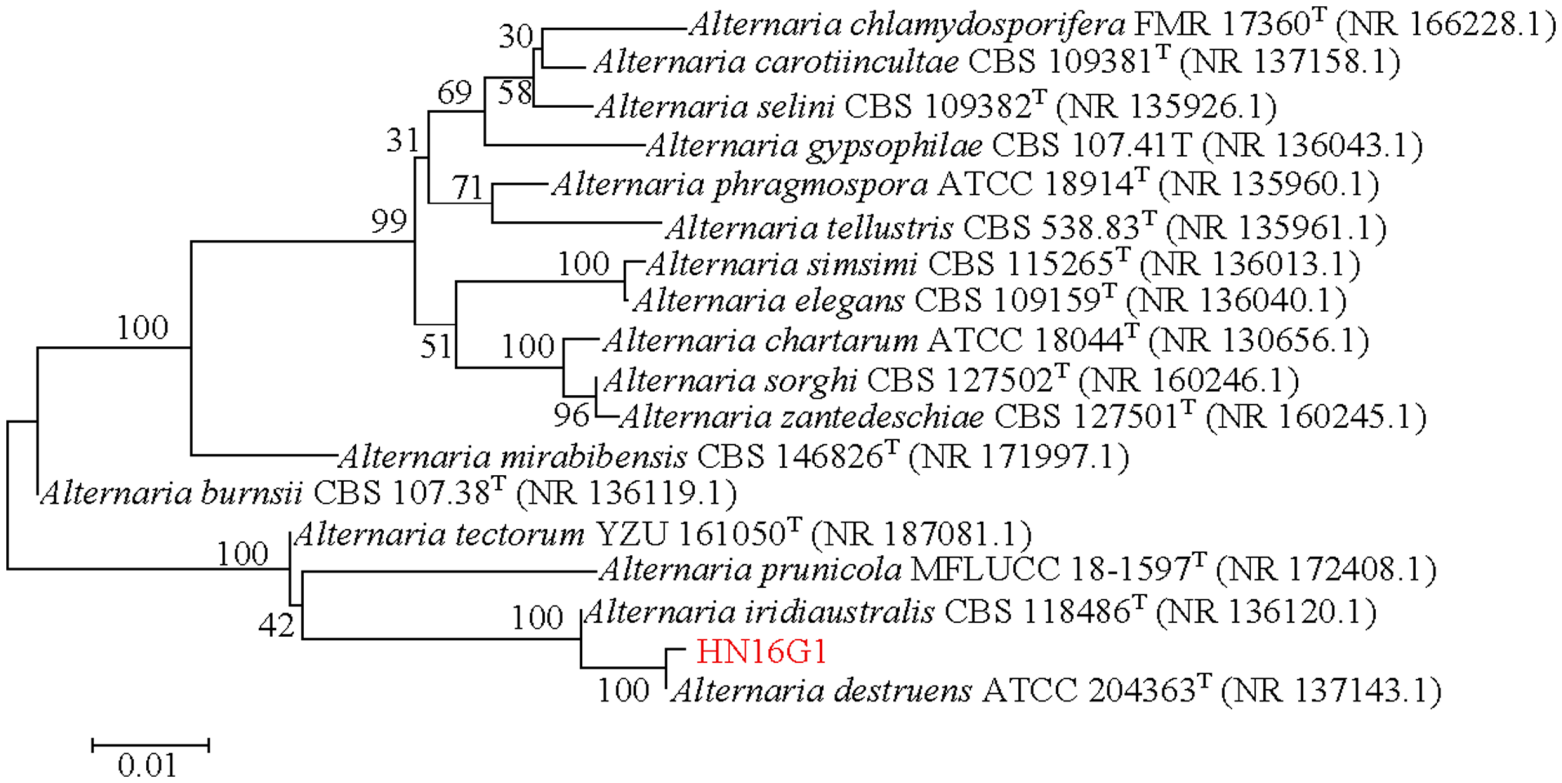
Neighbor-joining phylogenetic tree of the internal transcribed spacer (ITS) sequence from dark septate endophyte HN16G1.

To further clarify the taxonomic status of the HN16G1, the ITS region was amplified using the universal primers ITS1 and ITS4. Bidirectional sequencing yielded a 549 bp ITS sequence for strain HN16G1 (Accession No. PZ039114). Based on NCBI BLAST results, ITS sequences of 17 *Alternaria* type strains showing high homology with HN16G1 were selected to construct a NJ phylogenetic tree (Figure 6). HN16G1 clustered within the same clade as the type strain *Alternaria destruens* ATCC 204363, with a bootstrap value of 100, indicating a high degree of sequence homology between the two strains (Figure 6). Accordingly, HN16G1 was identified as *Alternaria destruens*.

### 3.4 Salt tolerance and IAA production capacity of HN16G1

**Figure 7:**
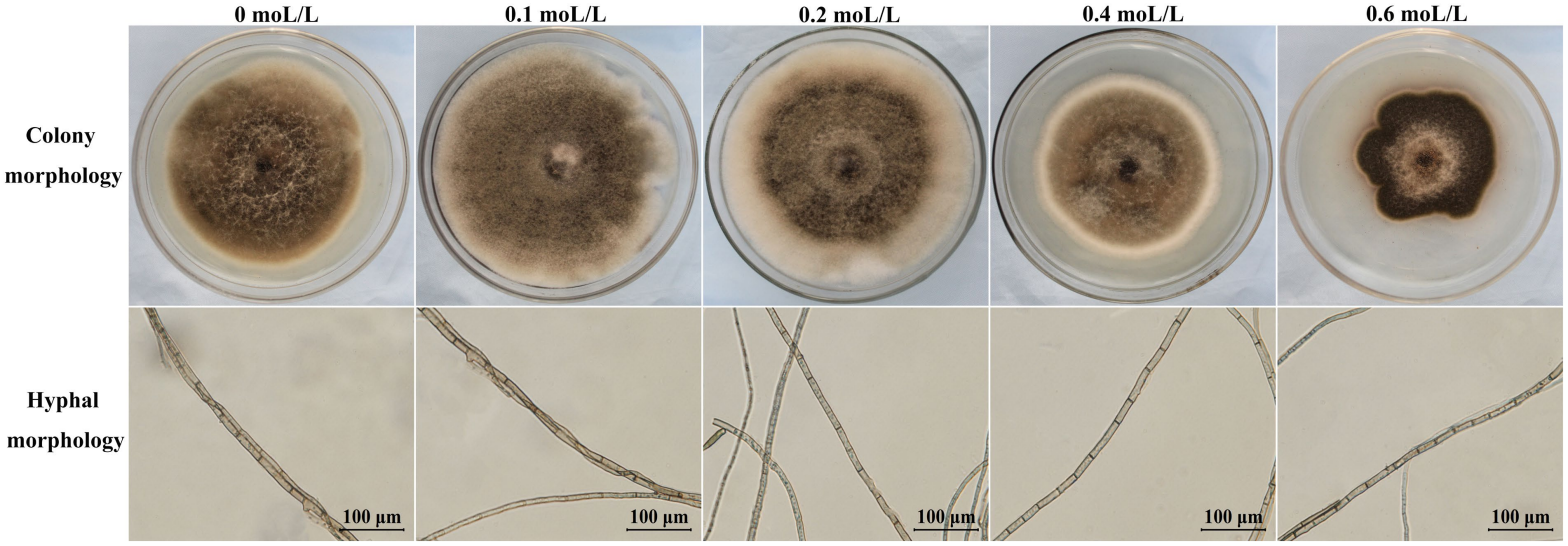
Effects of different salt concentrations on the colony and mycelial morphology of strain HN16G1.

**Figure 8:**
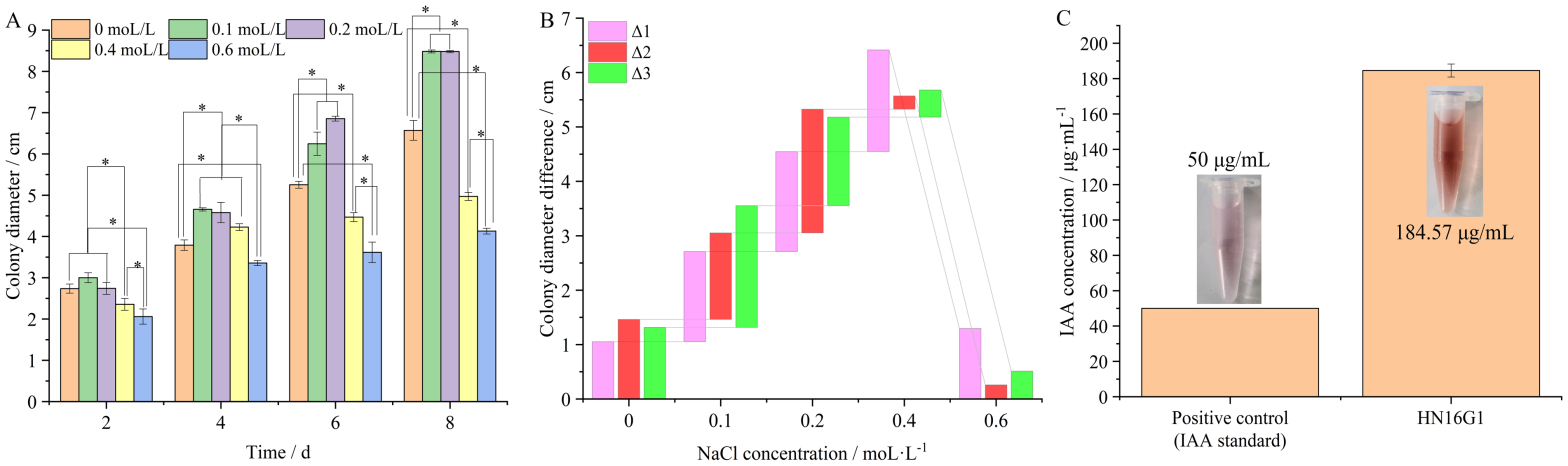
Growth response of the dark septate endophyte HN16G1 to salt stress **(A,B)** and the indole-3-acetic acid (IAA) synthesis capacity of stain HN16G1 **(C)**.

The colony morphology and microscopic characteristics of HN16G1 under different concentrations of NaCl stress are shown in Figure 7. In terms of colony diameter, when the NaCl concentration increased to 0.4 mol/L, the growth of the strain began to be inhibited, and the colony color gradually lightened, indicating that sporulation and pigment synthesis were significantly impeded. When the NaCl concentration was further increased to 0.6 mol/L, although growth inhibition was more pronounced, the colony color deepened, indicating that the strain may enhance its stress resistance under high-salt conditions by substantially producing spores and pigments. From the perspective of mycelial micromorphology, the hyphal septal distance initially increased and then decreased with increasing NaCl concentration, reaching a maximum at 0.1 mol/L and a minimum at 0.6 mol/L. A relatively low salt concentration (0.1 mol/L) may stimulate metabolic activity and promote hyphal elongation, thereby increasing the septal distance, reflecting an “overcompensation” effect in response to mild environmental changes (Mert and Ekmekçi, 1987; Pérez-Llano et al., 2020). Under high-salt stress (0.6 mol/L), the osmotic pressure increases significantly, and the strain may increase the number of septa (i.e., shorten the septal distance) to compartmentalize the cytoplasm and limit the spread of damaged or aging regions, thereby protecting overall hyphal survival—a self-defense mechanism under high-salt conditions (Kralj Kunčič et al., 2010; Jiménez-Gómez et al., 2020; Vangalis et al., 2020; Danilova et al., 2022). With respect to growth rate, the strain grew relatively rapidly at salt concentrations of 0.1–0.2 mol/L. After exposure to 0.4 mol/L, the growth rate gradually slowed except during the first 4 days (Δ1), and it decreased markedly at 0.6 mol/L, indicating that an appropriate salt concentration can promote the growth of HN16G1 (Figures 7, 8A,B). The IAA production capacity of HN16G1, determined using the Salkowski colorimetric method, revealed IAA secretion up to 184.57 μg/mL. These results indicated that HN16G1 possesses a strong ability to synthesize IAA, which may represent one of the important mechanisms underlying its plant growth-promoting effect (Figure 8C).

### 3.5 Effects of HN16G1 on drought tolerance of sorghum seedlings

The results of root irrigation inoculation with HN16G1 on the drought tolerance of sorghum seedlings showed that, under drought stress at 40% field capacity, compared with the control (without HN16G1 inoculation), an inoculation concentration of 5.0 × 10^5^ CFU/mL significantly increased the emergence rate and promoted the growth of seedlings across five sorghum varieties (Table 1); however, varietal differences in response were observed. Among the five varieties, HN16G1 exhibited the most pronounced growth-promoting effects on “Hongnuo 16” and “Jinnuo 3.” Under drought stress, these two varieties showed no significant difference in emergence rate, plant height, root length, stem diameter, and shoot fresh weight compared with that under the well-watered treatment. Colonization data under the same conditions further confirmed that “Hongnuo 16” had the highest colonization rate (43.05 ± 5.59 a) and intensity (8.71 ± 0.99 a), while “Jinnuo 3” also exhibited a relatively high colonization rate (37.22 ± 3.13 b) and comparable intensity (9.21 ± 0.73 a) (Supplementary Table S4). Although the emergence rates of the remaining three varieties recovered to levels comparable to those in the well-watered treatment under HN16G1 mediation, their growth phenotypes still differed from those under normal watering (Table 1). Consistently, “Jinza 22,” “Jinza 2001,” and “Jinbainuo 1” exhibited significantly lower colonization rates (27.50 ~ 31.11 c) with “Jinza 2001” showing the lowest colonization intensity (7.75 ± 1.23 b) (Supplementary Table S4). These findings indicate that the responsiveness to HN16G1 under drought stress varies among sorghum varieties, suggesting that the inoculation concentration for “Jinza 22,” “Jinza 2001,” and “Jinbainuo 1” requires further optimization.

**Table 1:** Effects of root drenching inoculation with strain HN16G1 on sorghum emergence rate and seedling growth under drought stress at 40% field capacity.

Sorghum	Treatment	Emergence rate (%)	Plant height (cm)	Root length (cm)	Stem diameter (cm)	Fresh weight (g)		Fresh weight (g)	
						Aboveground part	Underground part	Aboveground part	Underground part
Jin za 22	CK1	55.56 ± 6.80d	17.06 ± 1.06i	10.30 ± 0.71j	0.70 ± 0.04e	0.29 ± 0.03f	0.07 ± 0.02j	0.06 ± 0.02h	0.03 ± 0.01f
	CK2	79.17 ± 4.56ab	26.40 ± 0.88a	17.11 ± 0.69cd	0.79 ± 0.02d	0.40 ± 0.04b	0.12 ± 0.02def	0.11 ± 0.02bc	0.07 ± 0.02bc
	HN16G1	83.33 ± 5.27a	22.34 ± 1.69c	14.56 ± 0.47f	0.87 ± 0.05b	0.39 ± 0.02bc	0.10 ± 0.01fghi	0.11 ± 0.02bcd	0.06 ± 0.01cd
Hong nuo 16	CK1	62.50 ± 4.57c	19.21 ± 0.71fg	15.78 ± 0.9e	0.80 ± 0.04cd	0.33 ± 0.02de	0.09 ± 0.02hi	0.09 ± 0.01defg	0.04 ± 0.01ef
	CK2	79.17 ± 4.56ab	23.98 ± 0.84b	18.19 ± 0.45b	0.85 ± 0.03bc	0.42 ± 0.03ab	0.15 ± 0.01ab	0.12 ± 0.02ab	0.07 ± 0.01b
	HN16G1	81.94 ± 3.40a	23.06 ± 1.78bc	18.26 ± 0.43b	0.86 ± 0.03b	0.39 ± 0.02bc	0.13 ± 0.01cde	0.10 ± 0.02bcde	0.06 ± 0.01cd
Jin nuo 3	CK1	59.72 ± 3.40cd	18.03 ± 0.32hi	14.16 ± 0.34f	0.78 ± 0.04d	0.25 ± 0.03g	0.11 ± 0.02efgh	0.09 ± 0.02defg	0.04 ± 0.01ef
	CK2	76.39 ± 6.27ab	22.82 ± 0.91c	12.61 ± 0.48h	0.85 ± 0.03bc	0.35 ± 0.05cd	0.17 ± 0.01a	0.13 ± 0.01a	0.07 ± 0.01b
	HN16G1	77.78 ± 8.61ab	21.07 ± 1.41d	20.07 ± 1.03a	0.91 ± 0.02a	0.44 ± 0.04a	0.15 ± 0.01abc	0.11 ± 0.02bc	0.06 ± 0.01cd
Jin bainuo 1	CK1	56.94 ± 8.19cd	15.38 ± 0.45j	10.63 ± 0.31ij	0.64 ± 0.02f	0.29 ± 0.04f	0.08 ± 0.02ij	0.06 ± 0.02h	0.04 ± 0.01ef
	CK2	73.61 ± 6.27b	19.34 ± 0.59ef	13.40 ± 0.67 g	0.69 ± 0.06e	0.40 ± 0.04ab	0.12 ± 0.03defg	0.10 ± 0.02bcde	0.07 ± 0.02bc
	HN16G1	76.39 ± 6.27ab	18.27 ± 0.50gh	11.23 ± 0.45i	0.71 ± 0.06e	0.33 ± 0.05de	0.11 ± 0.03efghi	0.09 ± 0.02efg	0.05 ± 0.01de
Jin za 2001	CK1	62.50 ± 4.57c	14.83 ± 0.43j	13.26 ± 0.63gh	0.76 ± 0.04d	0.25 ± 0.02g	0.07 ± 0.03j	0.07 ± 0.01gh	0.04 ± 0.01ef
	CK2	79.17 ± 4.56ab	22.39 ± 1.07c	17.46 ± 0.74c	0.85 ± 0.03bc	0.35 ± 0.02cd	0.13 ± 0.03bcd	0.10 ± 0.02cdef	0.09 ± 0.01a
	HN16G1	81.94 ± 6.27a	20.34 ± 0.47de	16.53 ± 0.42d	0.77 ± 0.04d	0.30 ± 0.03ef	0.10 ± 0.03ghi	0.08 ± 0.01fg	0.06 ± 0.01bc

CK1 represents 40% field capacity without inoculation, and CK2 represents 70% field capacity without inoculation. Data in the table are the mean values of pot experiments conducted in 2023 and 2024. Different lowercase letters in the same column indicate significant differences (*P* < 0.05).

### 3.6 Effects of HN16G1 on the tolerance of sorghum seedlings to low-nutrient stress

Under the low-fertility conditions employed in this experiment, the emergence rates in CK1 (without HN16G1 inoculation) were significantly lower than those under normal fertilization for all varieties except “Jinza 2001.” Inoculation with HN16G1 under low fertility conditions significantly increased the emergence rates of “Hongnuo 16,” “Jinnuo 3,” “Jinbainuo 1,” and “Jinza 22.” Moreover, compared with those in CK2 (normal fertilization control), no significant differences were observed in the phenotypic traits, such as plant height, root length, stem diameter, and shoot fresh weight, across the tested varieties following HN16G1 inoculation (Table 2). Under the same low-nutrient stress, colonization data indicated that “Hongnuo 16” exhibited the highest colonization rate (38.33 ± 5.95 a) and intensity (9.76 ± 1.51 a), while “Jinnuo 3” also showed a relatively high rate (37.22 ± 5.09 ab) and comparable intensity (9.10 ± 0.90 ab) (Supplementary Table S4). Among the five varieties evaluated under low fertility conditions with HN16G1 mediation, “Hongnuo 16” and “Jinnuo 3” exhibited the most favorable growth phenotypes, indicating that HN16G1 possesses stronger affinity for these two varieties compared with that for the others (Table 2). Consistently, the remaining three varieties (“Jinza 22,” “Jinbainuo 1,” and “Jinza 2001”) exhibited lower colonization rates (33.06 ~ 34.44) with “Jinza 2001” showing the lowest colonization intensity (8.68 ± 0.84 b) (Supplementary Table S4).

**Table 2:** Effects of root drenching inoculation with strain HN16G1 on sorghum emergence rate and seedling growth under low-nutrient stress.

Sorghum	Treatment	Emergence rate (%)	Plant height (cm)	Root length (cm)	Stem diameter (cm)	Fresh weight (g)		Fresh weight (g)	
						Aboveground part	Underground part	Aboveground part	Underground part
Jin za 22	CK1	56.94 ± 6.27gh	18.70 ± 1.11e	11.08 ± 1.06i	0.75 ± 0.05e	0.32 ± 0.05de	0.11 ± 0.01de	0.06 ± 0.02f	0.04 ± 0.01g
	CK2	86.28 ± 5.64a	26.30 ± 0.70a	17.54 ± 0.75ab	0.82 ± 0.02abcd	0.42 ± 0.05bc	0.18 ± 0.02a	0.11 ± 0.02 cd	0.07 ± 0.01abc
	HN16G1	83.33 ± 5.27ab	23.03 ± 0.52bc	14.41 ± 0.54ef	0.86 ± 0.06ab	0.44 ± 0.03b	0.15 ± 0.02bc	0.10 ± 0.02 cd	0.06 ± 0.01cde
Hong nuo 16	CK1	59.72 ± 8.20fgh	19.97 ± 0.50d	12.64 ± 0.50gh	0.68 ± 0.04f	0.46 ± 0.06b	0.08 ± 0.01fg	0.10 ± 0.01 cd	0.04 ± 0.01fg
	CK2	79.17 ± 4.56abc	23.92 ± 1.02b	16.12 ± 0.82cd	0.78 ± 0.03cde	0.56 ± 0.04a	0.14 ± 0.01c	0.14 ± 0.03ab	0.08 ± 0.01ab
	HN16G1	77.78 ± 4.30abc	23.45 ± 0.78bc	17.87 ± 1.41ab	0.78 ± 0.06de	0.57 ± 0.04a	0.16 ± 0.02abc	0.13 ± 0.03bc	0.06 ± 0.01cde
Jin nuo 3	CK1	68.06 ± 8.19def	17.17 ± 1.06f	13.17 ± 1.24fg	0.66 ± 0.06f	0.37 ± 0.04cd	0.07 ± 0.01gh	0.05 ± 0.01f	0.04 ± 0.01fg
	CK2	80.28 ± 5.91ab	22.59 ± 0.72c	15.99 ± 1.89d	0.84 ± 0.02abc	0.56 ± 0.06a	0.15 ± 0.02bc	0.14 ± 0.02ab	0.07 ± 0.01bcde
	HN16G1	80.55 ± 4.30ab	22.70 ± 0.65c	17.43 ± 0.39bc	0.87 ± 0.05a	0.52 ± 0.06a	0.14 ± 0.02cd	0.09 ± 0.02de	0.06 ± 0.01def
Jin bainuo 1	CK1	52.78 ± 8.61h	15.45 ± 0.76 g	11.25 ± 1.23hi	0.66 ± 0.05f	0.31 ± 0.04de	0.08 ± 0.02fgh	0.05 ± 0.01f	0.04 ± 0.01fg
	CK2	65.28 ± 13.35efg	19.80 ± 0.96d	14.02 ± 1.15efg	0.81 ± 0.06bcde	0.42 ± 0.06bc	0.17 ± 0.02ab	0.10 ± 0.02 cd	0.07 ± 0.01abcd
	HN16G1	65.28 ± 9.74efg	20.52 ± 0.46d	15.32 ± 1.09de	0.84 ± 0.03ab	0.40 ± 0.03bc	0.12 ± 0.02de	0.10 ± 0.03de	0.06 ± 0.01cde
Jin za 2001	CK1	70.33 ± 5.37cde	15.15 ± 1.06g	11.72 ± 0.73hi	0.76 ± 0.02e	0.31 ± 0.03e	0.06 ± 0.02 h	0.07 ± 0.01ef	0.05 ± 0.01efg
	CK2	76.39 ± 9.74bcd	22.73 ± 1.29c	18.96 ± 1.53a	0.85 ± 0.03ab	0.40 ± 0.03bc	0.13 ± 0.02 cd	0.16 ± 0.02a	0.08 ± 0.01ab
	HN16G1	76.39 ± 3.40bcd	18.80 ± 0.92e	18.56 ± 1.79ab	0.82 ± 0.02abcd	0.37 ± 0.05 cd	0.10 ± 0.02ef	0.14 ± 0.02ab	0.08 ± 0.01a

CK1 represents low-nutrient stress without inoculation, and CK2 represents normal fertilization without inoculation. Data in the table are the mean values of pot experiments conducted in 2023 and 2024. Different lowercase letters in the same column indicate significant differences (*P* < 0.05).

### 3.7 Interaction between colonization rate and intensity of HN16G1 and sorghum seedling phenotype

**Figure 9:**
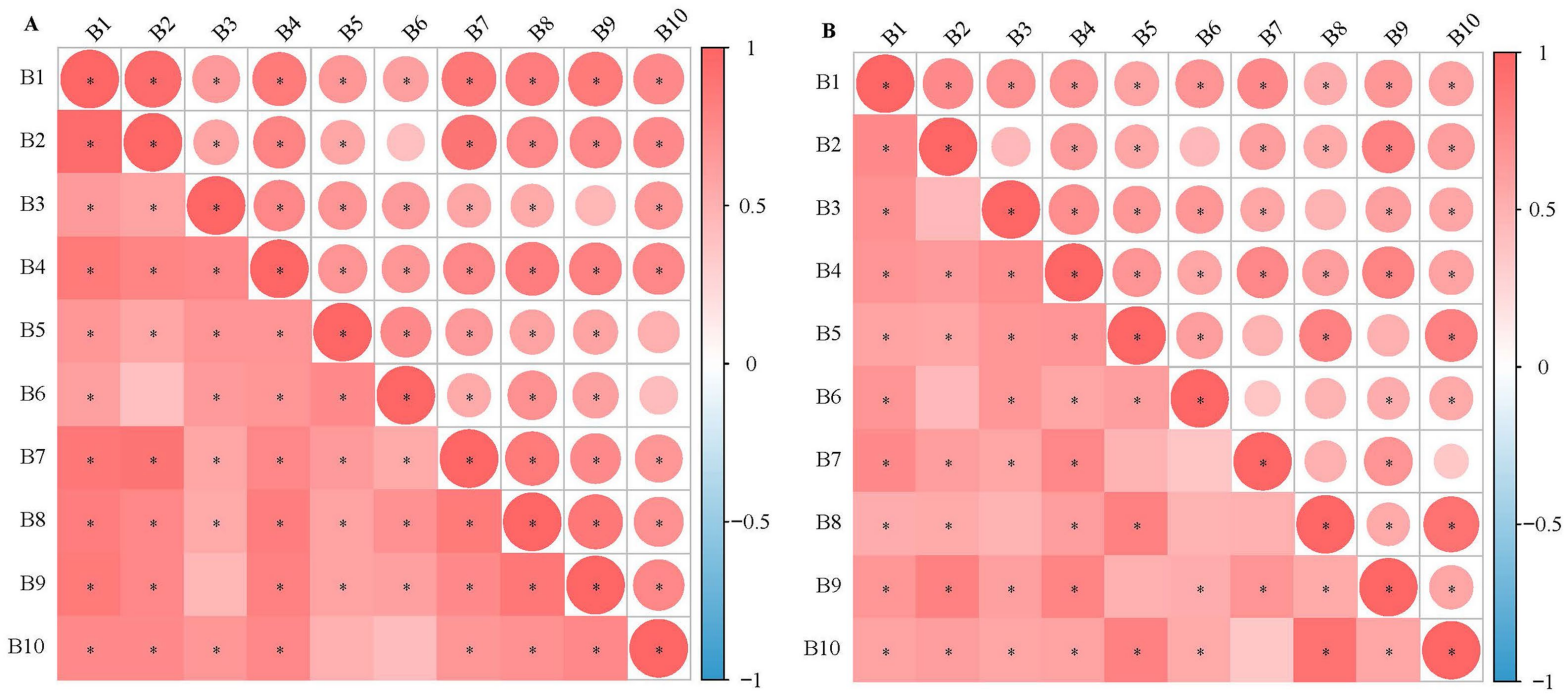
Correlation between the rate and intensity of colonization of strain HN16G1 in sorghum root tissues and the emergence rate and growth phenotype of sorghum. Panel **(A)** represents drought stress, and Panel **(B)** represents low-nutrient stress. B1–B10 denote colonization rate, colonization intensity, emergence rate, plant height, root length, stem diameter, aboveground fresh weight, belowground fresh weight, aboveground dry weight, and belowground dry weight, respectively. “*” indicates a significant correlation at the 0.05 level.

Correlation analysis between the colonization rate (B1) and colonization intensity (B2) of HN16G1 (Supplementary Table S4) and sorghum growth phenotypes revealed that under both drought stress and low-fertility stress, both the colonization parameters (B1 and B2) were positively correlated with the emergence rate and phenotypic traits of sorghum. Moreover, significant correlations were observed with phenotypic indicators, such as plant height (B4), root length (B5), and biomass (B7–B10), whereas no significant correlation was found with stem diameter (Figures 9A,B). These results indicate that enhancing the colonization ability of HN16G1 in sorghum roots under drought and low-fertility stress can significantly improve the drought tolerance and low-fertility resistance of sorghum seedlings.

## 4 Discussion

DSE represent a functionally significant group within the plant root microecosystem, which can regulate plant growth, development, and stress responses by establishing symbiotic relationships with their hosts, and their community composition and distribution are influenced by differences in the host genotype (Rodriguez et al., 2009; Hughes et al., 2020; de Oliveira and Pereira, 2025). The application potential of DSE in stress-resistant cultivation has been demonstrated for various crops, especially in marginal land production, where they can enhance host nutrient-use efficiency and stress tolerance to achieve high yield and better quality (Hou et al., 2021; Huertas et al., 2024; Lalthazuali et al., 2025). In this study, we systematically investigated the distribution patterns and colonization characteristics of culturable DSE in the rhizosphere soil and root tissues of 29 sorghum varieties, as well as the regulatory effects of the dominant strain HN16G1 on sorghum resistance to drought and low-fertility stress. The findings provide key theoretical insights into the symbiotic mechanisms operative between sorghum and DSE, which should help improve stress tolerance in sorghum.

A total of 306 fungal isolates were obtained from 29 sorghum cultivars for the first time. DSE accounted for 30.72% of the isolates and were widely distributed across 24 cultivars, which is reflective of high abundance and broad host adaptability of DSE in the sorghum root microecosystem. This distribution pattern is consistent with observations in other gramineous crops, such as wheat, maize (Bokati et al., 2016; Wang et al., 2016; Chen et al., 2023), and *Ammopiptanthus mongolicus* (Li et al., 2018). Notably, significant variation in the number of culturable DSE was observed among different sorghum cultivars (Bock et al., 2025). The highest number of DSE isolates (18) was obtained from the cultivar Hongnuo 16, whereas no DSE were detected in some cultivars. These results confirm the selective effect of host genotype on the assembly of DSE community, aligning with findings that not all plant species or cultivars could be colonized by DSE, as certain genotypes exhibit a natural absence of such fungal associations (Jumpponen and Trappe, 1998; Mandyam and Jumpponen, 2005; Mandyam et al., 2013). Additional, this study revealed clear differences in colonization efficiency among the DSE strains across cultivars, suggesting that host genotype had a stronger regulatory influence on DSE colonization than soil ecological conditions, which is in concordance with previous studies (Han et al., 2021; Zuo et al., 2022; Li S. et al., 2024). Host plants can modulate the rhizosphere microenvironment through root exudates (e.g., phenolic compounds, sugars), thereby affecting the colonization and proliferation of microbes (Wu et al., 2013; Saravesi et al., 2014; Wu et al., 2017; Sun et al., 2021). Hongnuo 16 is hypothesized to provide more favorable colonization conditions for DSE by secreting specific chemical signaling substances—a hypothesis that requires further validation through metabolomic analysis of root exudates. Additionally, the high rate of DSE detection in sorghum roots observed in this study (82.76% of cultivars harbored DSE) exceeded the 50.5–62.5% colonization rate previously reported for inoculated sorghum (Zhang et al., 2017). This discrepancy may be attributable to regional differences in soil physicochemical properties, climatic conditions, and cultivar-specific genetic backgrounds.

We identified 30 morphotypes of DSE, among which, the percentage of isolates related to the genus *Alternaria* was as high as 46.67%. Morphotypes, such as HN16G1 and JNH10T1, were able to colonize multiple sorghum cultivars, with HN16G1 isolated from eight of the tested cultivars. This indicates that *Alternaria* represents a dominant DSE group in sorghum roots. Moreover, DSE belonging to the genus *Alternaria* were isolated from across different ecological regions and soil types. Similar results have been reported previously; for example, *Alternaria alternata* was the predominant DSE in the rhizosphere of *Salvia miltiorrhiza* (Han et al., 2025), *Alternaria* sp. was predominant in the rhizosphere of xerophytic plants in the desert region of Northwest China (Zuo et al., 2022), and *Alternaria chartarum* was the predominant DSE in the rhizosphere of *Artemisia ordosica* (Li et al., 2022b). However, the other predominant DSE had been found in other crops such as *Stagonosporopsis* (He et al., 2021), *Paraphoma chrysantemicola*, *Acrocalymma vagum* (Li et al., 2022b), *Poaceascoma helicoides*, and *Setophoma terrestris* (Xu et al., 2024). Furthermore, different DSE strains exhibited varying degrees of host specificity. For instance, HN11G1 could only infect three sorghum cultivars, whereas strains, such as HN16G1 and JL3G3, were capable of infecting ten different cultivars. This variation in host specificity is similar to the differences in wheat, blueberry, and tomato (Li et al., 2022a; Li S. et al., 2024; Feng et al., 2025). Such differences may stem from compatibility variations in the signal recognition systems between strains and their hosts. The specific mechanisms underlying this phenomenon require further investigation using genomic and transcriptomic technologies.

Analysis of colonization characteristics revealed that certain strains exhibited high colonization rate but low colonization intensity, reflecting the heterogeneity in the colonization traits of the DSE strains. This observation aligns with the strain-specific differences in colonization reported by Mandyam et al. (2013) in their study on DSE in *Arabidopsis thaliana*, highlighting the complexity of DSE–host interactions. Among the DSE strains examined, HN16G1, JL3G3, and HZ23G1 demonstrated high rates and intensity of colonization. In particular, HN16G1 achieved colonization rates exceeding 50% across 10 sorghum cultivars, with colonization intensity greater than 16%, indicating excellent symbiotic compatibility. These results indicate that HN16G1 may establish a stable symbiotic relationship with sorghum through the formation of more efficient colonization structures, such as dark septate hyphae and microsclerotia, and that this high colonization capability serves as a prerequisite for its regulatory role in stress resistance. Future studies could employ confocal laser scanning microscopy to observe the colonization sites and pathways of HN16G1 colonization within sorghum roots, thereby clarifying the morphological features of its symbiotic interface.

Stress tolerance experiments confirmed that HN16G1 inoculation significantly improved drought and low-fertility tolerance of sorghum seedlings. The cultivars Hongnuo 16 and Jinnuo 3 exhibited the most pronounced responses to HN16G1, with their emergence rates and growth phenotypes under drought or low-fertility conditions recovering to levels comparable to those under normal irrigation and fertilization. These results are consistent with correlation analysis, which revealed that the rate and intensity of HN16G1 colonization were significantly positively correlated with sorghum growth indicators, such as plant height, root length, and biomass. This indicates that the colonization capability of DSE directly determines its stress-alleviating effects. This observation aligns with the finding that *Alternaria* CTW-9 significantly enhances drought tolerance in potatoes, as reported by Bai et al. (2023), reflecting the strain-specific nature of DSE–host interactions under stress conditions. Moreover, these results support the hypothesis proposed by Barresi et al. (2022), stating that DSE can promote the growth of sorghum by regulating the transformation of soil phosphorus fractions. However, the present study further clarifies the positive correlation between DSE colonization rate and low-fertility tolerance, while also highlighting the regulatory role of host genotype variation in mediating this effect. From a physiological perspective, DSE may enhance stress tolerance of sorghum through the following mechanisms: (1) expanding the root absorption area to improve water and nutrient (e.g., nitrogen, phosphorus) uptake and utilization (Chen et al., 2023; Bock et al., 2025); (2) synthesizing osmoregulatory substances, such as abscisic acid and proline, to enhance the osmotic stress tolerance of host (Chen et al., 2023; Zuo et al., 2025); and (3) improving the rhizosphere microenvironment, for example, by modulating soil enzyme activity, to increase nutrient availability (Spagnoletti et al., 2017; Bi et al., 2025a; Guo et al., 2025; Wang Z. et al., 2025). The differential responses of sorghum cultivars to HN16G1 observed in this study further illustrate the coevolutionary relationship between host genotype and DSE. The high affinity of HN16G1 for cultivars Hongnuo 16 and Jinnuo 3 likely contributes to their superior growth performance under stress conditions.

Compared with previous studies, we systematically elucidated the differences in the distribution and colonization characteristics of DSE among sorghum cultivars, identified *Alternaria* as the dominant group, and isolated a strain, HN16G1, with broad-spectrum colonization ability and efficient stress-tolerance regulatory functions. This provides a high-quality microbial resource for developing stress-resistant inoculants for sorghum. Relative to the foundational survey of sorghum DSE distribution reported previously (Becerra et al., 2023), this study expands the range of cultivars examined and provides an in-depth analysis of the link between DSE colonization traits and host stress responses. Compared with the preliminary investigation by Barresi et al. (2022) on DSE-mediated phosphorus uptake regulation in sorghum, this study clarifies the broad applicability of specific dominant strains and the cultivar-specific response patterns, offering more precise theoretical support for targeted development of inoculant.

However, this study has some limitations. First, the investigation of the DSE community was restricted to culturable isolates, leaving non-culturable members uncharacterized. Second, stress-tolerance experiments were conducted only at the seedling stage; thus, the effects of strain HN16G1 on stress resistance and yield in mature sorghum plants remain to be validated. Third, the molecular mechanisms underlying HN16G1-mediated enhancement of sorghum stress tolerance were not examined in depth.

Future research should address these gaps through several approaches. Field trials are needed to evaluate the effects of HN16G1 on grain yield in mature sorghum under drought and low-fertility conditions, as well as to determine optimal inoculation timing. In addition, high-throughput sequencing should be employed to comprehensively characterize DSE community composition in the sorghum rhizosphere across different cultivars and growth stages. Integrated transcriptomic and metabolomic analyses will further elucidate the molecular mechanisms underlying HN16G1-mediated stress tolerance. Moreover, optimization of inoculation concentration and application methods is required, along with evaluation under additional stress conditions (e.g., saline–alkali stress) to support the development of cultivar-specific inoculants. Notably, previous studies indicate that DSE exhibit strong salt tolerance, and that moderate salinity can promote their growth; therefore, extending research on HN16G1 under saline–alkali stress is of considerable practical significance.

## 5 Conclusion

This study demonstrates that Alternaria spp. are the dominant culturable DSE in sorghum roots and identifies a broad-spectrum, highly compatible strain, Alternaria destruens HN16G1. This strain exhibits intrinsic salt tolerance—potentially associated with modulation of hyphal septation—and a high capacity for IAA production, enabling effective colonization across multiple sorghum varieties. Inoculation with HN16G1 significantly enhances seedling emergence and biomass under both drought and low-nutrient conditions, with growth-promoting effects positively correlated with colonization intensity, indicating that colonization strength may serve as a predictor of field performance. Notably, HN16G1 forms stable symbiotic associations with all tested varieties (colonization rate > 50%, intensity > 16%), highlighting its broad-spectrum compatibility. Overall, HN16G1 represents a promising multi-functional DSE resource for improving sorghum productivity under nutrient-limited and water-deficit conditions, offering a sustainable biological strategy to reduce reliance on chemical inputs in dryland agriculture.

## Data Availability

The datasets presented in this study can be found in online repositories. The names of the repository/repositories and accession number(s) can be found in the article/Supplementary material.

## Footnotes

1
https://cloud.metware.cn/
