# Supplementary material for: Isolation and identification of dark septate endophytes from sorghum roots and the effects of Alternaria destruens HN16G1 on drought and low-nutrient tolerance in sorghum seedlings
Source: Front Microbiol. 2026 May 22;17:1829731. doi: 10.3389/fmicb.2026.1829731 (PMC13236965; doi:10.3389/fmicb.2026.1829731)
Supplement: Supplementary file 1 [file Table_1.docx]

Supplementary Material

# Supplementary Figures and Tables

## Supplementary Figures

**
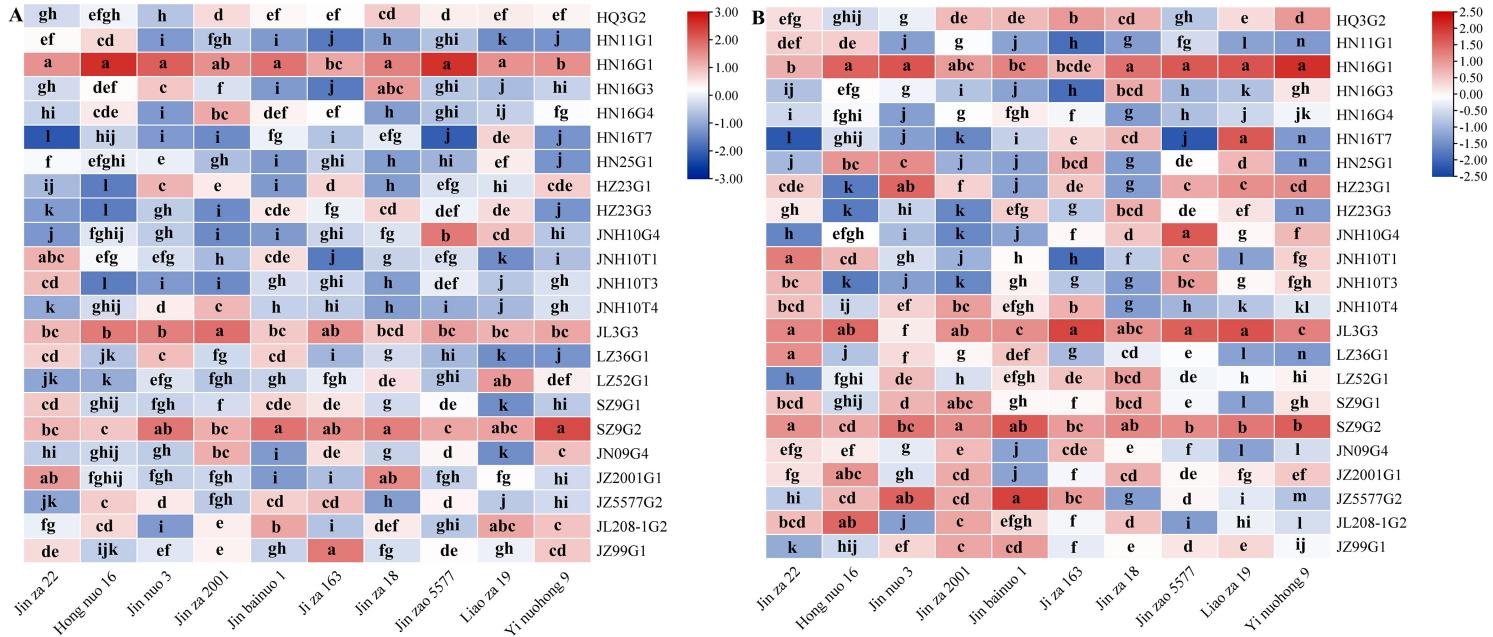
**

**Supplementary Figure 1.**Significance analysis of differences in the rate and intensity of colonization of 23 dark septate endophytic fungi across 10 different sorghum varieties.

Note: Different lowercase letters in the same column indicate significant differences among different fungal strains at *P* < 0.05. A represents drought stress, and B represents low-nutrient stress.

## Supplementary Tables

**Supplementary Table 1.** Number of rhizosphere soil fungi and root endophytic fungi in different sorghum varieties

| Sorghum varieties | Number of fungi isolated from rhizosphere soil | Number of endophytic fungi isolated from sorghum roots | Number of dark septate fungi | Sorghum varieties | Number of fungi isolated from rhizosphere soil | Number of endophytic fungi isolated from sorghum roots | Number of dark septate fungi |
| --- | --- | --- | --- | --- | --- | --- | --- |
| Hong nuo 8 | 6 | 3 | 0 | Jin za 22 | 7 | 5 | 3 |
| Hong nuo 11 | 4 | 3 | 3 | Jin za 2001 | 8 | 4 | 8 |
| Hong nuo 13 | 5 | 3 | 0 | Jin za 28 | 9 | 5 | 3 |
| Hong nuo 16 | 14 | 10 | 18 | Jin za 51 | 5 | 2 | 1 |
| Hong nuo 18 | 4 | 3 | 0 | Jin za 21-1 | 8 | 4 | 2 |
| Hong nuo 19 | 7 | 4 | 0 | Jin zao 5577 | 4 | 3 | 1 |
| Hong nuo 25 | 4 | 2 | 4 | Jin zao 5564 | 4 | 2 | 0 |
| Han qing 3 | 9 | 4 | 4 | Jin za 99 | 6 | 1 | 1 |
| Hui za 23 | 12 | 5 | 5 | Jin liang 3 | 11 | 8 | 3 |
| Jin nuohong 10 | 15 | 6 | 5 | Jin bainuo 1 | 3 | 2 | 3 |
| Sui za 9 | 8 | 2 | 2 | Jin liang 208 | 6 | 2 | 4 |
| Liao nuo 10 | 5 | 2 | 4 | Jin nuoliang 9 | 7 | 4 | 5 |
| Liao nuo 11 | 6 | 3 | 4 | Jin nuoliang 10 | 5 | 4 | 2 |
| Liao za 36 | 4 | 3 | 2 | Jin nuo 3 | 6 | 4 | 5 |
| Liao za 52 | 7 | 4 | 2 |  |  |  |  |

**Supplementary Table 2.** Occurrence and distribution of 30 dark septate fungal isolates among different sorghum varieties.

| Isolated number | Sorghum varieties |
| --- | --- |
| HQ3G2 | “Han qing 3” “Jin za 2001” “Hong nuo 16” “Hui za 23” |
| HN11G1 | 3 “Hong nuo 11” “Han Qing 3” “Jin Za 2001” |
| HN16G1 | “Hong nuo 16” “Jin za 2001” “Bai nuo 1” “Liao nuo 10” “Jin Za 22” “Jin nuo 3” “Hong nuo 25” “Hui Za 23” |
| HN16T2 | “Hong nuo 16” |
| HN16G3 | “Hong nuo 16” “Jin nuo liang 9” |
| HN16G4 | “Hong nuo 16” “Han qing 3” “Hong nuo 11” “Jin liang 208” “Jin nuo Liang 9” |
| HN16T7 | “Hong nuo 16” “Jin nuo 3” “Bai nuo 1”“Liao nuo 11” “Jin liang 208” |
| HN25G1 | “Hong nuo 25” “Hong nuo 16” |
| HN25T1 | “Hong nuo 25” “Hong nuo 16” |
| HN265T2 | “Liao nuo 10” “Hong nuo 16” |
| HZ23G1 | “Hui Za 23” |
| HZ23G3 | “Hui Za 23” “Jin nuo hong 10” “Jin nuo 9” |
| JNH10G4 | “Jin nuo hong 10” “Hong nuo 16” “Jin nuo liang 10” “Jin za 22” “Jin nuo 3” |
| JNH10T1 | “Jin nuo hong 10” “Hong nuo 16” “ Hui za 23” “Han qing 3” “Jin nuo liang 10” “Jin liang 3” |
| JNH10T3 | “Jin nuo hong 10” “Bai nuo 1” “Hong nuo 25” |
| JNH10T4 | “Jin nuo hong 10” “Liao nuo 10” “Liao nuo 11” “Hong nuo 16” |
| JL3G3 | “Jin liang 3” “Jin za 2001” “Jin za 22” “Jin za 28” “Jin liang 208” |
| JL3T10 | “Jin liang 3” |
| JZ272T2 | “Liao nuo 11” “Hong nuo 16” |
| JZ28T5 | “Jin za 28” |
| JZ51T1 | “Jin za 51” |
| LZ36G1 | “Liao za 36” “Liao za 52” “Hong nuo 16” |
| LZ52G1 | “Liao za 52” |
| SZ9G1 | “Sui za 9” “Hong nuo 16” “Jin za 2001” “Jin za 28” |
| SZ9G2 | “Sui za 9” “Hong nuo 16” “Jin nuo 3” “Jin za 2001” |
| JN09G4 | “Jin nuo liang 9” “Jin za 2001” “Hong nuo 11” “Liao nuo 10” “Liao nuo 11” |
| JZ2001G1 | “Jin za 2001” “Jin nuo 3” “Jin nuo liang 9” “Hong nuo 16” |
| JZ5577G2 | “Jin zao 5577” “Liao za 36” “Jin za 21-1” |
| JL208-1G2 | “Jin liang 208” “Jin za 2001” “Jin za 21-1” “Hong nuo 16” |
| JZ99G1 | “Jin za 99” |

**Supplementary Table 3.** Symbiotic compatibility of 23 dark septate endophytic fungi with 10 commercially dominant sorghum varieties.

| Isolated number | Sorghum varieties | | | | | | | | | |
| --- | --- | --- | --- | --- | --- | --- | --- | --- | --- | --- |
|  | Jin za 22 | Hong nuo 16 | Jin nuo 3 | Jin za 2001 | Jin bainuo 1 | Ji za 163 | Jin za 18 | Jin zao 5577 | Liao za 19 | Yi nuohong 9 |
| HQ3G2 | + | + | + | + | + | + | + | + | + | + |
| HN11G1 | + | + | —— | —— | —— | —— | —— | + | —— | —— |
| HN16G1 | + | + | + | + | + | + | + | + | + | + |
| HN16G3 | + | + | + | —— | —— | —— | + | + | + | + |
| HN16G4 | + | + | —— | + | + | + | —— | + | + | + |
| HN16T7 | —— | + | —— | + | + | + | + | —— | + | —— |
| HN25G1 | + | + | + | —— | + | + | —— | + | + | —— |
| HZ23G1 | + | —— | + | —— | —— | + | —— | + | + |  |
| HZ23G3 | + | —— | + | + | —— | + | + | + | + | —— |
| JNH10G4 | + | + | + | —— | —— | + | + | + | + | + |
| JNH10T1 | + | + | + | + | + | —— | + | + | —— | + |
| JNH10T3 | + | —— | —— | + | + | + | —— | + | + | + |
| JNH10T4 | + | + | + | + | + | + | —— | + | + | + |
| JL3G3 | + | + | + | + | + | + | + | + | + | + |
| LZ36G1 | + | + | + | + | + | + | + | + | —— | —— |
| LZ52G1 | + | + | + | + | + | + | + | + | + | + |
| SZ9G1 | + | + | + | + | + | + | + | + | —— | + |
| SZ9G2 | + | + | + | + | + | + | + | + | + | + |
| JN09G4 | + | + | + | —— | —— | + | + | + | —— | + |
| JZ2001G1 | + | + | + | —— | + | + | + | + | + | + |
| JZ5577G2 | + | + | + | + | —— | + | —— | + | + | + |
| JL208-1G2 | + | + | —— | + | + | + | + | + | + | + |
| JZ99G1 | + | + | + | + | + | + | + | + | + | + |

Note: “— ” indicates no colonization; “+ ” indicates colonization.

**Supplementary Table 4.** Colonization Rate/ Intensity of strain HN16G1 in different sorghum varieties under drought and low-nutrient stress.

| Sorghum | Treatment | Colonization Rate (%) | | Colonization Intensity (%) | |
| --- | --- | --- | --- | --- | --- |
|  |  | Drought stress at 40% field capacity | Low-nutrient stress | Drought stress at 40% field capacity | Low-nutrient stress |
| Jin za 22 | HN16G1 | 31.11±4.99 c | 34.17±5.71 ab | 8.71±0.99 ab | 9.05±0.78 ab |
| Hong nuo 16 | HN16G1 | 43.05±5.59 a | 38.33±5.95 a | 9.77±0.96 a | 9.76±1.51 a |
| Jin nuo 3 | HN16G1 | 37.22±3.13 b | 37.22±5.09 ab | 9.21±0.73 a | 9.10±0.90 ab |
| Jin bainuo 1 | HN16G1 | 27.50±9.65 c | 34.44±3.28 ab | 8.90±2.15 a | 9.98±1.04 a |
| Jin za 2001 | HN16G1 | 30.28±9.04 c | 33.06±6.11 b | 7.75±1.23 b | 8.68±0.84 b |

Note: Data in the table are the mean values of pot experiments conducted in 2023 and 2024. Different lowercase letters in the same column indicate significant differences (*P* < 0.05). “——” indicates that the treatment was not detected. CK groups were not inoculated and showed no colonization.
